# 1,25-dihydroxyvitamin D3 signaling-induced decreases in IRX4 inhibits NANOG-mediated cancer stem-like properties and gefitinib resistance in NSCLC cells

**DOI:** 10.1038/s41419-020-02908-w

**Published:** 2020-08-20

**Authors:** Zhirong Jia, Yameng Zhang, Aiwen Yan, Meisa Wang, Qiushuang Han, Kaiwei Wang, Jie Wang, Chen Qiao, Zhenzhen Pan, Chuansheng Chen, Dong Hu, Xuansheng Ding

**Affiliations:** 1 School of Basic Medicine and Clinical Pharmacy, China Pharmaceutical University, 211198 Nanjing, China; 2 Department of Pharmacy, the First Affiliated Hospital of Xinjiang Medical University, 830054 Urumqi, China; 3 Precision Medicine Laboratory, School of Basic Medicine and Clinical Pharmacy, China Pharmaceutical University, 211198 Nanjing, China; 4 Key Laboratory of Industrial Dust Prevention and Control & Occupational Safety and Health of the Ministry of Education, Medical School, Anhui University of Science and Technology, 232001 Huainan, China

**Keywords:** Cancer therapy, Cancer

## Abstract

Recent studies have demonstrated that acquisition of cancer stem-like properties plays an essential role in promoting epidermal growth factor receptor-tyrosine kinase inhibitors (EGFR-TKIs) resistance in non-small cell lung cancer (NSCLC); however, how to regulate cancer stem-like properties and EGFR-TKI resistance is largely unclear. In this study, we discovered that increased iroquois-class homeodomain protein 4 (IRX4) was related to gefitinib resistance in NSCLC cells. Knockdown of IRX4 inhibited cell proliferation, sphere formation, and the expression of CD133, ALDH1A1, NANOG, Sox2 and Notch1, and the transcriptional activity of NANOG promoter. IRX4 overexpression increased the protein level of NANOG and CD133 in PC-9 cells. Combination of knocking-down IRX4 with gefitinib increased cell apoptosis and decreased cell viability and the expression of p-EGFR and NANOG in PC-9/GR cells. IRX4 knockdown in a PC-9/GR xenograft tumor model inhibited tumor progression and the expression of NANOG and CD133 more effectively than single treatment alone. Knockdown of NANOG inhibited the expression of CD133 and restored gefitinib cytotoxicity, and NANOG overexpression-induced cancer stem-like properties and gefitinib resistance could be obviously reversed by knocking-down IRX4. Further, we found that 1,25-dihydroxyvitamin D3 (1,25(OH)_2_D_3_) reduced obviously the expression of IRX4 and NANOG by inhibiting the activation of TGF-β1/Smad3 signaling pathway; moreover, combination of 1,25(OH)_2_D_3_ and gefitinib decreased cell viability and proliferation or tumor progression and the expression of IRX4 and NANOG compared with single treatment alone both in PC-9/GR cells and in a PC-9/GR xenograft tumor model. These results reveal that inhibition of IRX4-mediated cancer stem-like properties by regulating 1,25(OH)_2_D_3_ signaling may increase gefitinib cytotoxicity. Combination therapy of gefitinib and 1,25(OH)_2_D_3_ by targeting IRX4 and NANOG, could provide a promising strategy to improve gefitinib cytotoxicity.

## Introduction

Lung cancer has been noted due to the increasing rate of morbidity and mortality worldwide^1^. Many patients with lung adenocarcinoma (LUAD), a major subtype of lung cancer^2^, harbor mutations in the epidermal growth factor receptor (EGFR) in their cancer tissues and initially react well to molecular targeted drugs such as gefitinib, which inhibits the EGFR-tyrosine kinase^3,4^. However, acquired drug resistance inevitably occurs within 10–14 months, leading to poor prognosis^5,6^. Several mechanisms of acquired resistance to EGFR-tyrosine kinase inhibitors (EGFR-TKIs) have been elucidated, mainly including the mutation of *EGFR* T790M, *MET* and *HER2* amplification^7^. Whereas, underlying resistance mechanism remains undefined in a significant percentage of patients. Therefore, it is of great significance to investigate potential mechanisms and alternative strategies for reversing gefitinib resistance or enhancing its efficacy.

Growing evidence revealed that stem cell-like properties were involved in EGFR-TKI resistance. Non-small cell lung cancer (NSCLC) cells developed cancer stem cell-like properties after acquiring resistance to afatinib^8^. In addition, the delayed development of cancer stem-like cells was accompanied with reduced tumor burden and improved recurrence free survival as well as overall survival in xenograft models of EGFR-mutant NSCLC cells^9^. Further, acquisition of stemness phenotype after the emergence of EGFR-TKI resistance enhanced tumor metastasis in lung cancer^10^. Consequently, during a long-term exposure to TKIs, the appearance and enrichment of cancer stem-like cells may be one of the causes for acquired resistance^11^. Nevertheless, how to regulate the stem-like properties deserves further study.

Iroquois-class homeodomain protein 4 (IRX4) is a protein that in humans is encoded by the *IRX4* gene. The analysis showed upregulated expression of IRX4 in lung tissues of NSCLC patients and a negative association between IRX4 expression and survival rate of NSCLC patients^12^. Further, genome-wide identification of NSCLC suggested that IRX4, functioning as a carcinogenic transcription factor, was positively correlated with cell proliferation. Despite these advances, the role of IRX4 in NSCLC as well as in EGFR-TKI resistance remains largely unknown.

The IRX-family genes participate in the development of embryonic tissues in a variety of modes by encoding IRX proteins, and appear to play different roles at different stages of the embryo^13,14^. Studies have shown that IRX4^+^mouse embryonic cells have multi-directional differentiation potential and high proliferative capacity^15^, and *IRX4a* regulates the expression of the *sox2* gene, both in the neural plate and in progenitor cells of the lateral line system^16^. This indicates that IRX4-positive cells have differentiation potential and characteristics of stem-like cell. However, whether IRX4 regulate the cancer stem-like properties of EGFR-TKI resistant cells needs further study.

Pre-clinical models support the idea that the active metabolite of vitamin D3, 1,25-dihydroxyvitamin D3 (1,25(OH)_2_D_3_) inhibits lung cancer growth^17^. Of note, NSCLC cells with an EGFR mutation also respond well to 1,25(OH)_2_D_3_, and 1,25(OH)_2_D_3_/erlotinib combination increased erlotinib cytotoxicity in both the erlotinib-sensitive HCC827 cell line and the erlotinib-resistant H1975 cell line^18^. However, how 1,25(OH)_2_D_3_ regulate EGFR-TKI sensitivity is unknown. It has been reported that 1,25(OH)_2_D_3_ inhibited cancer cell stemness^19^. This led us to speculate that 1,25(OH)_2_D_3_ may inhibit EGFR-TKI resistance by reducing cancer cell stemness. In this study, the role of IRX4 in regulating EGFR-TKI resistance and cancer stem-like properties, and the effects of 1,25(OH)_2_D_3_ on regulating IRX4-mediated cancer cell stemness and EGFR-TKI resistance, were investigated.

## Results

### IRX4 expression is upregulated by gefitinib exposure

**Fig. 1:**
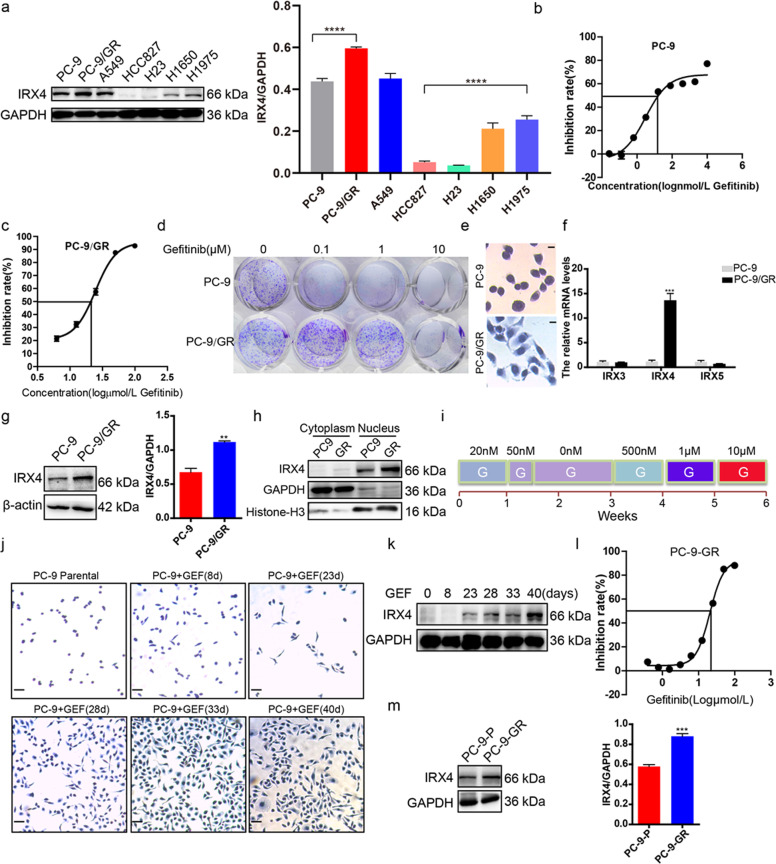
IRX4 expression is upregulated by gefitinib exposure. **a** IRX4 protein level in LUAD cells was detected by western blotting. The IRX4 expression level relative to GAPDH was quantified with a ChemiScope analysis (mean ± SD; *n* = 3; ****P < 0.0001). **b**–**c** PC-9 and PC-9/GR cells were respectively stimulated with various concentrations of gefitinib for 48 h. MTT assay were carried out to determine the IC50 value against gefitinib. **d** PC-9 and PC-9/GR cells were treated with various concentrations of gefitinib for 10 days. The colony formation was detected by crystal violet staining. **e** HE staining was performed to detect the cell morphology of PC-9 and PC-9/GR cells (Scale bar: 100 μm; original magnification: ×100; representative images from three experiments). **f** The mRNA levels of *IRX3*, *IRX4* and *IRX5* were evaluated by QRT-PCR (mean ± SD; *n* = 6; ***P < 0.001). **g** IRX4 protein levels in PC-9 and PC-9/GR cells were assessed by western blotting. The relative intensity of IRX4 was evaluated by ChemiScope analysis software (mean ± SD; *n* = 3; **P < 0.01). **h** Nuclear and cytoplasmic fractions of PC-9 and PC-9/GR cells were prepared, and IRX4 expression was detected by western blotting. GAPDH and Histone-H3 were used respectively as cytoplasm and nucleus loading control. **i** The steps of inducing gefitinib-resistant PC-9 cells: PC-9 cells were first treated with gefitinib at the concentration of 20 nM for 1 week. A small amount of remaining cells were then treated for another 2 days with a concentration of 50 nM which was sufficient to kill nearly all parental cells. The remaining few cells were cultured continuously in the absence of gefitinib for 2 weeks. Then the cells were sequentially treated with gefitinib at the concentration of 500 nM for 1 week, 1 μM for another 1 week and 10 μM for the last 1 week. **j** The PC-9 cells morphology of different time points stimulated with gefitinib were determined by HE staining (Scale bar: 100 μm; original magnification: ×100; representative images from three experiments). **k** IRX4 protein levels at day 8, 23, 28, 33 and 40 were determined by western blotting. **l** MTT assay was carried out to determine IC50 value against gefitinib of PC-9-GR cell. **m** Western blot analysis of IRX4 protein levels in PC-9 and PC-9-GR cells. The relative intensity of IRX4 was evaluated by ChemiScope analysis software (mean ± SD; *n* = 3; ***P < 0.001).

We found that IRX4 was widely expressed in LUAD cells, IRX4 expression was significantly higher in PC-9/GR cells than that in PC-9 cells, and was also obviously higher in H1975 cells than that in HCC827 cells (Fig. 1a). The paired high (PC-9/GR) and low (PC-9) IRX4-expressing cell lines were used for further studies. The detection of IC50 values against gefitinib and colony formation confirmed that PC-9 was gefitinib-sensitive and PC-9/GR was gefitinib-resistant (Fig. 1b–d). We also found that the morphology of PC-9 and PC-9/GR cells was different (Fig. 1e). Then, the upregulation of IRX4 in PC-9/GR cells was confirmed by QRT-PCR and western blotting, however, the mRNA levels of IRX-family members such as *IRX3* and *IRX5* had no significant change (Fig. 1f, g). The IRX4 was mainly expressed in the nucleus and the nuclear expression of IRX4 was higher in PC-9/GR cells than that in PC-9 cells (Fig. 1h), indicating IRX4 functions in the nucleus. Then, a rapid method inducing gefitinib-resistant PC-9 cells (PC-9-GR) was established (Fig. 1i). The changing cell morphology was recorded at indicated time points (Fig. 1j). The IRX4 expression was gradually increasing with the prolongation of gefitinib exposure (Fig. 1k). IC50 values against gefitinib of PC-9-GR cells was about 20 μM (Fig. 1l), and IRX4 expression in PC-9-GR cells was obviously elevated than in PC-9 cells (Fig. 1m).

### Downregulation of IRX4 expression enhances the gefitinib cytotoxicity of PC-9/GR cells

**Fig. 2:**
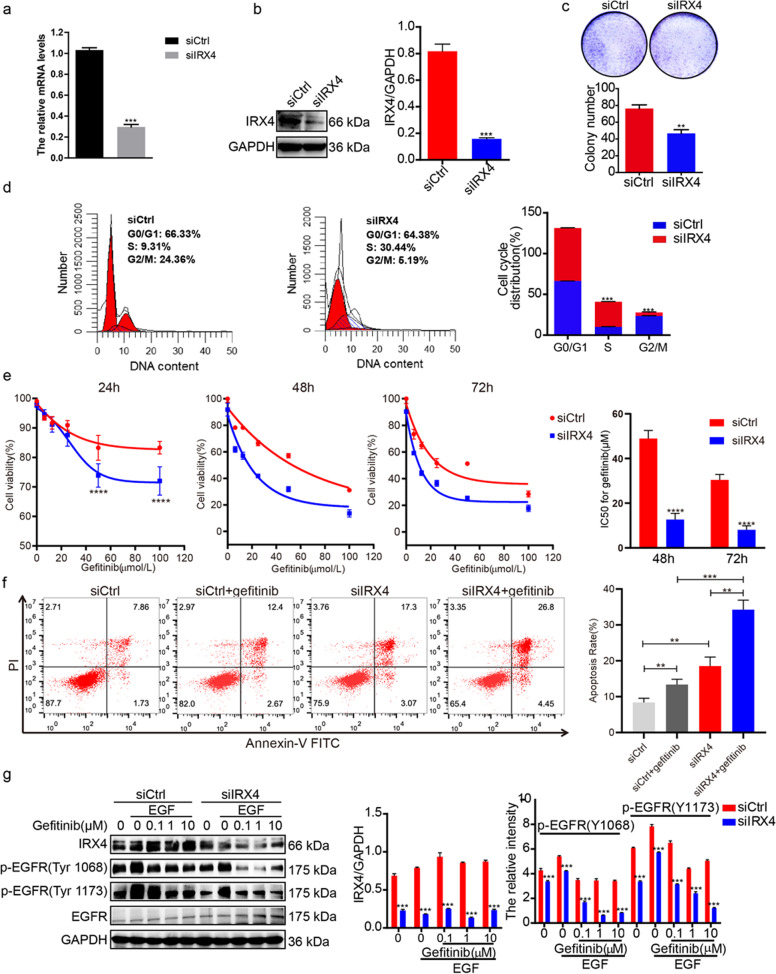
Downregulation of IRX4 expression enhances the gefitinib cytotoxicity of PC-9/GR cells. **a**–**b** PC-9/GR cells were transfected with siCtrl or siIRX4, after 48 h, the silence efficiency of IRX4 were determined by QRT-PCR and western blotting (mean ± SD; *n* = 6; ***P < 0.001). **c** Silencing IRX4 inhibited the clonogenic activity of PC-9/GR (mean ± SD; *n* = 3; **P < 0.01). **d** The cells were transfected with siIRX4, and harvested after 48 h, then fixed with 70% cold ethanol, incubated with the mixture of RNase and propidium iodide (BD, Franklin Lake, NJ, USA) for 15 min at room temperature. Cell cycle distribution was analyzed by flow cytometry (Miltenyi Biotec) and ModFit LT 5.0 software (Verity Software House, Topsham, ME, USA) (mean ± SD; *n* = 3; ***P < 0.001). **e** siRNA was transfected, after 6 h, various concentration of gefitinib (0, 6.25, 12.5, 25, 50, 100 μmol/L were co-administered with siRNA respectively for 24, 48 and 72 h, then MTT assay was carried out to determine the cytotoxicity and the IC50 value against gefitinib was calculated (mean ± SD; *n* = 3; ****P < 0.0001). **f** An Annexi*n* V-FITC apoptosis kit (Vazyme, Nanjing, China) was used to determine the number of apoptotic cells according to the manufacturer’s instructions. The cells were sorted using a FACS flow cytometer (Miltenyi Biotec). The total apoptotic rates were assessed and analyzed by flow cytometry after siCtrl, siCtrl+geftinib, siIRX4 or siIRX4 + Gefitinib treatment for 48 h and the results were analyzed using FlowJo VX software (mean ± SD; *n* = 3; **P < 0.01, ***P < 0.001). The experiments were repeated three times. **g** siRNA was transfected for 48 h, then the cells were stimulated with EGF (50 ng/mL) for 5 min, and the protein were extracted, the expression of IRX4, p-EGFR (Y1068), p-EGFR (Y1173) and total EGFR were detected by western blotting. The relative intensity of each protein was evaluated by ChemiScope analysis software (mean ± SD; *n* = 3; ***P < 0.001).

As the results shown, IRX4 silence (Fig. 2a, b) prominently inhibited the colony formation of PC-9/GR cells (Fig. 2c), and led to cell cycle arrest at S phase (Fig. 2d). The detection of cell viability and apoptosis indicated that the combination of knocking-down IRX4 and gefitinib was more sensitive to gefitinib than the single treatment group (Fig. 2e, f). Furthermore, we found that combination group further reduced the expression of p-EGFR compared with the single treatment (Fig. 2g).

### Knocking-down IRX4 decreases NANOG expression and stemness of PC-9/GR cells

**Fig. 3:**
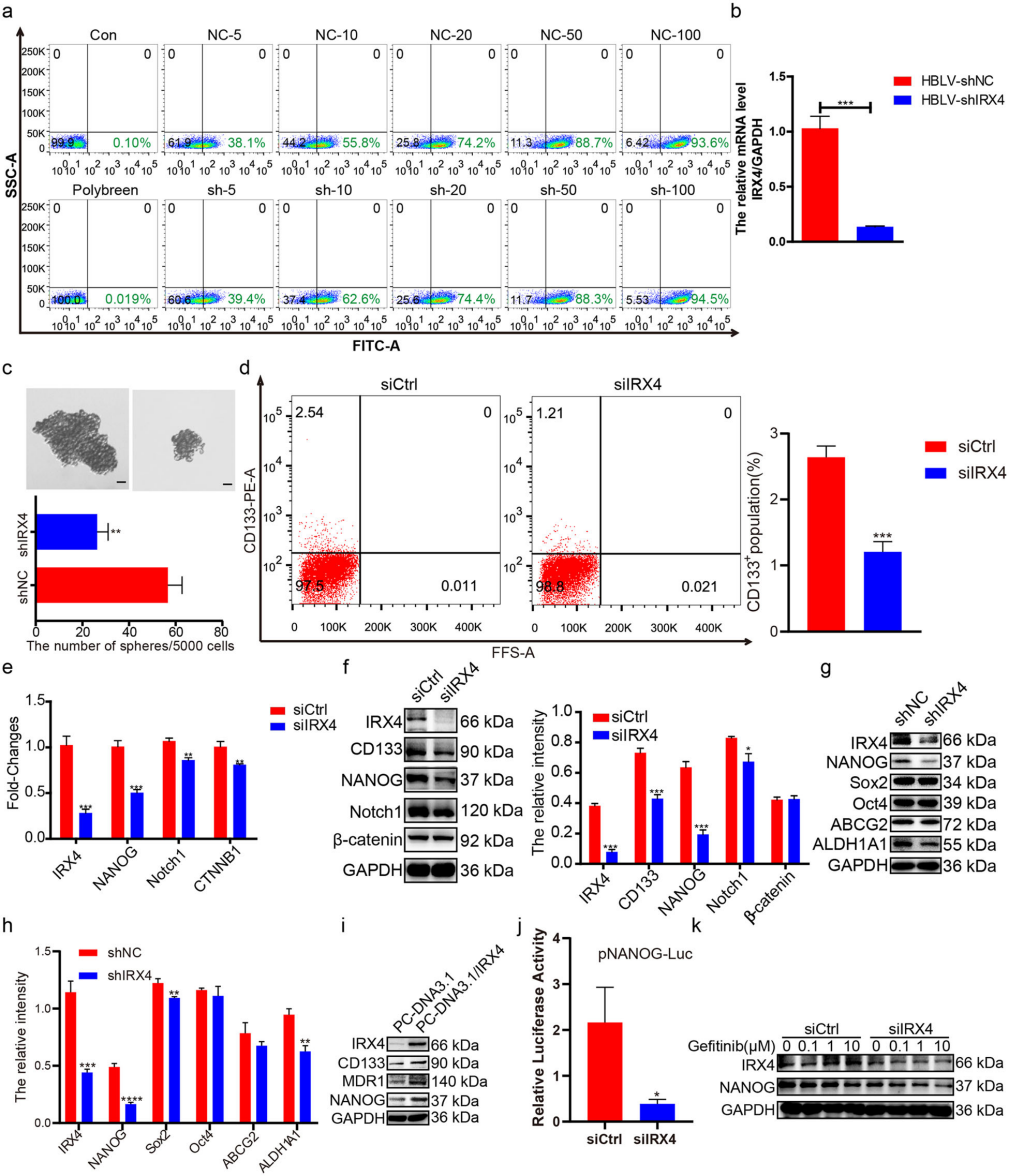
Inhibition of IRX4 decreases NANOG levels and stemness of NSCLC cells. **a** PC-9/GR cells were infected with lentivirus for 72 h. Note: Numbers represent MOI values; lentivirus concentration: 4.0 × 10^8^TU/mL. **b** PC-9/GR cells were infected with lentivirus for 48 h, then the total RNA was extracted, and mRNA level of IRX4 was detected by real-time PCR (mean ± SD; *n* = 6; ***P < 0.001). **c** A total of 5 × 10^3^ cells were seeded into 6-well ultra low-attachment plates (Corning, NY, USA) and incubated in DMEM/F12 (Biological Industries) supplemented with EGF(20 ng/mL), FGF-basic(20 ng/mL) and B27 (20 μL/mL) for two weeks. Cell spheres characterized by tight, spherical, non-adherent colonies of more than 90μm in diameter were observed and counted (Scale bar: 100 μm; original magnification: ×100; representative images from three experiments; mean ± SD; **P < 0.01). **d** The PC-9/GR cells were transfected with siIRX4 or siCtrl, after 48 h the percentage of CD133^+^ cells were analyzed by flow cytometry (mean ± SD; *n* = 3; ***P < 0.001). **e** PC-9/GR cells were transfected with control small interference RNA (siCtrl) or IRX4‐specific siRNA (siIRX4). After 48 h, the relative mRNA levels of *IRX4*, *NANOG*, *Notch1* and *CTNNB1* were measured by QRT‐PCR (mean ± SD; *n* = 6; **P < 0.01, ***P < 0.001). **f** PC-9/GR cells were transfected with siCtrl or siIRX4. After 48 h, western blotting was performed to detect the expression of IRX4, CD133, NONOG, Notch1, and β-catenin, and the relative intensity of these bands was analyzed (mean ± SD; *n* = 3; *P < 0.05, ***P < 0.001). The data were representatives of 3 independent experiments. **g**–**h** PC-9/GR cells were infected with lentivirus for 72 h, the expression of IRX4, NANOG, Sox2, Oct4, ABCG2 and ALDH1A1 was detected by western blotting, and the relative intensity of each protein was calculated and analyzed by ChemiScope analysis software (mean ± SD; *n* = 3; **P < 0.01, ***P < 0.001, ****P < 0.0001). **i** PC-9 cells were transfected with empty pcDNA3.1 vectors, or pcDNA3.1-IRX4 for 48 h. The protein levels of IRX4, CD133, MDR1 and NANOG were determined by western blotting. **j** NANOG transcriptional activity was measured after PC-9/GR cells were transfected with siIRX4 or siCtrl for 48 h. Results are shown as histogram showing quantitative values from triplicate experiments (mean ± SD; *n* = 3; *P < 0.05). **k** After transfetcted with siCtrl or siIRX4 for 6 h, PC-9/GR cells were treated with gefitinib (0.1, 1, 10μmol/L) for 48 h, and then western blot analysis was performed to assess the expression of IRX4 and NANOG.

Next, lentivirus-ShRNA-IRX4 carrying enhanced green fluorescent protein (ZsGreen) was generated, and the best infection conditions were determined. Both the group of shNC and shIRX4 had the similar infection efficiency (Fig. 3a), and the IRX4 expression was effectively knocked-down (Fig. 3b). IRX4 knockdown in PC-9/GR cells significantly inhibited sphere formation (Fig. 3c) and reduced the percentage of CD133^+^ cells (Fig. 3d). Moreover, IRX4 knockdown downregulated mRNA levels of stemness-related genes (*NANOG*, *Notch1* and *CTNNB1*) (Fig. 3e) and reduced protein levels of CD133, NANOG, Notch1, Sox2, and ALDH1A1, however, have no obvious effect on the expression of Oct4 and ABCG2 (Fig. 3f-h). Conversely, IRX4 overexpression increased the expression of CD133, MDR1, and NANOG (Fig. 3i). Moreover, IRX4 knockdown caused a significant impairment of NANOG transcriptional activity as measured by the pNANOG-Luc reporter activity (Fig. 3j). The combination of knocking-down IRX4 with various concentration of gefitinib reduced obviously the NANOG expression than that of transfecting control siRNA (Fig. 3k).

### NANOG mediates the regulation of IRX4 on cancer stem-like properties and gefitinib resistance

**Fig. 4:**
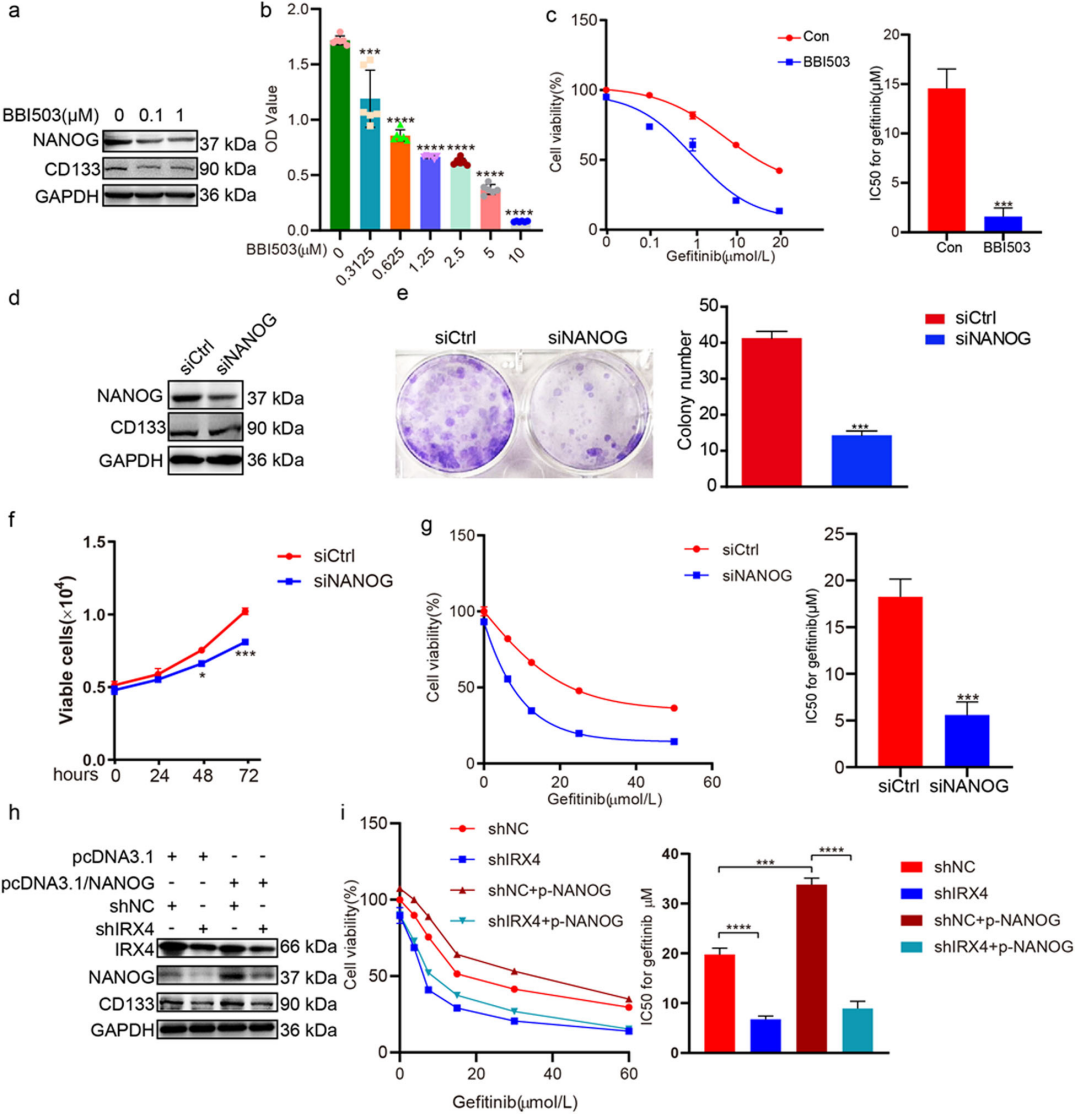
NANOG mediates the regulation of IRX4 on cancer stem-like properties and gefitinib resistance. **a** PC-9/GR cells were exposed to BBI503 (0.1 and 1μmol/L) for 48 h and then harvested for western blotting to detect the NANOG and CD133 expression. **b** Treatment with BBI503 at indicated concentrations for 48 hours suppressed the growth of PC-9/GR cells in a dose-dependent manner. ***P < 0.001, ****P < 0.0001. **c** Effect of combined treatment with BBI503 (0.1 μM) and gefitinib on growth of PC-9/GR cells was evaluated, and the IC50 against gefitinib was calculated (mean ± SD; *n* = 3; ***P < 0.001). **d** PC-9/GR cells were transfected with NANOG siRNA (siNANOG) or control siRNA (siCtrl). Total cell lysate proteins were extracted for western blot analysis of NANOG and CD133 expression using specific antibodies. **e**–**f** The detection of clonal formation and cell viability after silencing NANOG expression (mean ± SD; *n* = 3; ***P < 0.001). **g** PC-9/GR cells were transfected with siNANOG or siCtrl, after 6 h, the cells were exposed to various concentrations of gefitinib (0, 6.25, 12.5, 25, 50 μM) for another 48 h. Cell proliferation was determined by MTT assay, and the IC50 against gefitinib was calculated. Data represent the mean ± SD of three replicate determinations, ***P < 0.001. **h** PC-9/GR cells were infected with lentivirus carrying shIRX4 or shNC for 24 h, and then NANOG was overexpressed by transfecting with the pcDNA3.1-NANOG plasmid or the empty vector (pcDNA3.1) for another 48 h, and the expressions of IRX4, NANOG and CD133 were evaluated by western blotting. **i** PC-9/GR cells were infected with lentivirus carrying shIRX4 or shNC for 24 h, and then transfected with pcDNA3.1-NANOG plasmid or the empty vector, at 6 h post-transfection, the cells were treated with various concentrations of gefitinib (0, 3.75, 7.5, 15, 30, 60 μM) for another 48 h. Cell proliferation was determined by CCK8 assay, and the IC50 against gefitinib was calculated. Data represent the mean ± SD of three replicate determinations, ***P < 0.001, ****P < 0.0001.

We found that BBI503 (Amcasertib), which is a small-molecule multi-kinase inhibitor^20^, inhibited the NANOG and CD133 expression (Fig. 4a), and cell viability (Fig. 4b) in PC-9/GR cells. Moreover, the combination of BBI503 with various concentration of gefitinib decreased the cell viability significantly (Fig. 4c). By silencing NANOG, the expression of NANOG and CD133 in PC-9/GR cells was also reduced (Fig. 4d). Additionally, NANOG silence inhibited colony formation (Fig. 4e) and cell viability of PC-9/GR cells (Fig. 4f). NANOG silence combined with gefitinib significantly decreased the cell viability compared with gefitinib alone (Fig. 4g). Furthermore, increase of NANOG and CD133 expression, and gefitinib resistance induced by NANOG overexpression could be reversed by knocking-down IRX4 (Fig. 4h, i).

### In vivo antitumor activity of combined knocking-down IRX4 and gefitinib therapy

**Fig. 5:**
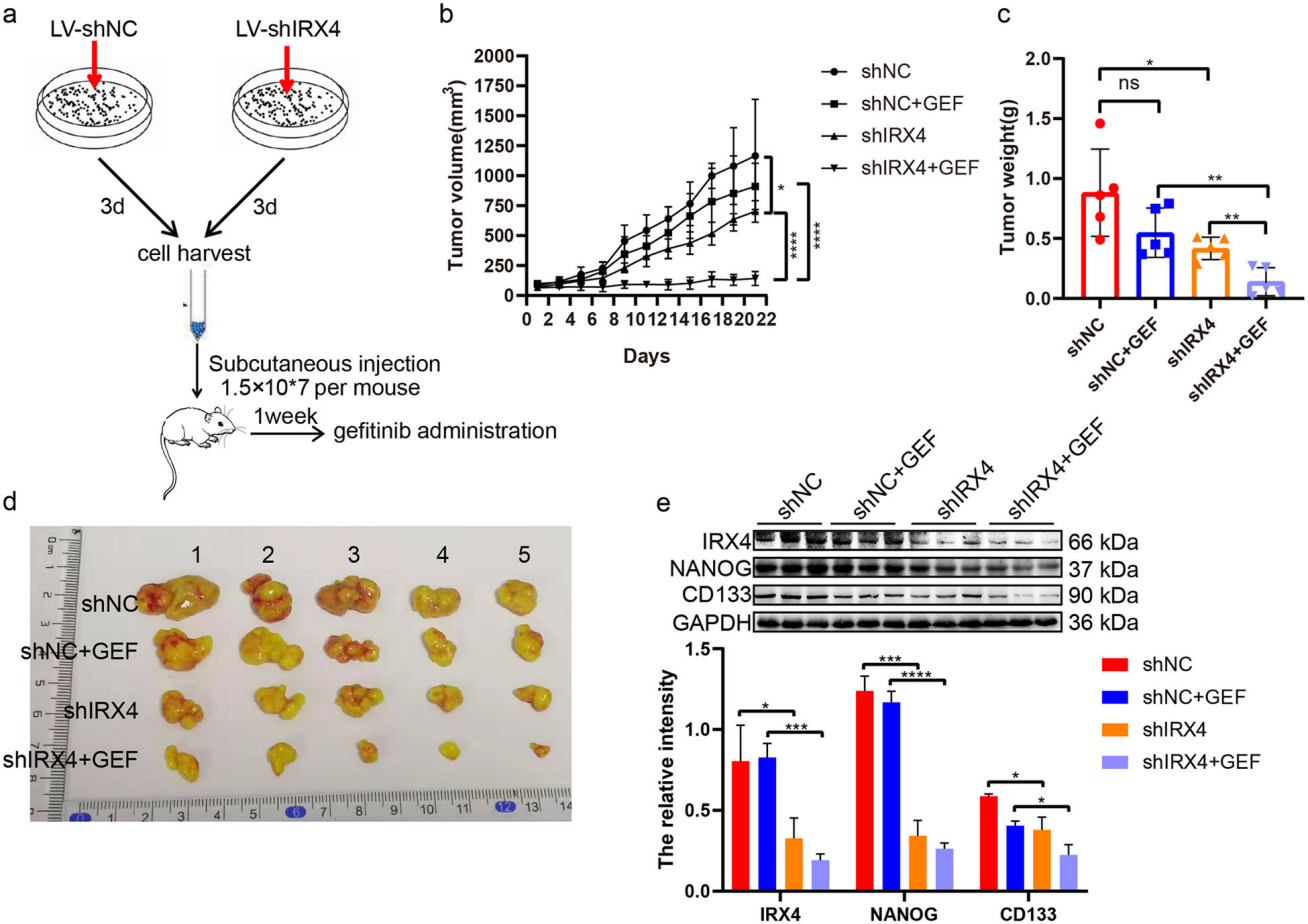
Downregulation of IRX4 increases gefitinib cytotoxicity and inhibits expression of NANOG and CD133 in a PC-9/GR xenograft mouse model. **a** Flow charts of the establishment of xenograft mouse model and gefitinib was administered during day 7 – day 21. **b**–**c** Tumor sizes and weight were presented as mean ± SD; *n* = 5; ns, not significant, *P < 0.05, **P < 0.01, ****P < 0.0001. **d** Macroscopic appearance of PC- 9/GR xenografts of each group. **e** Whole protein cell lysates were prepared randomly from 3 tumors per group for western blotting to detect the indicated proteins (mean ± SD; *P < 0.05, ***P < 0.001, ****P < 0.0001).

The establishment of the xenograft mice model was illustrated as shown in the figure (Fig. 5a). As expected, the combination of shIRX4 and gefitinib significantly suppressed tumor progression compared with any single treatment (Fig. 5b-d). Moreover, downregulation of IRX4 inhibited the expression of NANOG and CD133, and combination of knocking-down IRX4 and gefitinib further decreased significantly the expression of IRX4, NANOG and CD133 compared with administrating gefitinib alone (Fig. 5e). The effects of IRX4 downregulation increasing gefitinib cytotoxicity and inhibiting cancer stem-like properties are well demonstrated in a PC-9/GR xenograft mouse model.

### 1,25(OH)_2_D_3_ (1,25D) reduces IRX4 and NANOG expression by inhibiting TGF-β1/Smad3 signaling pathway

**Fig. 6:**
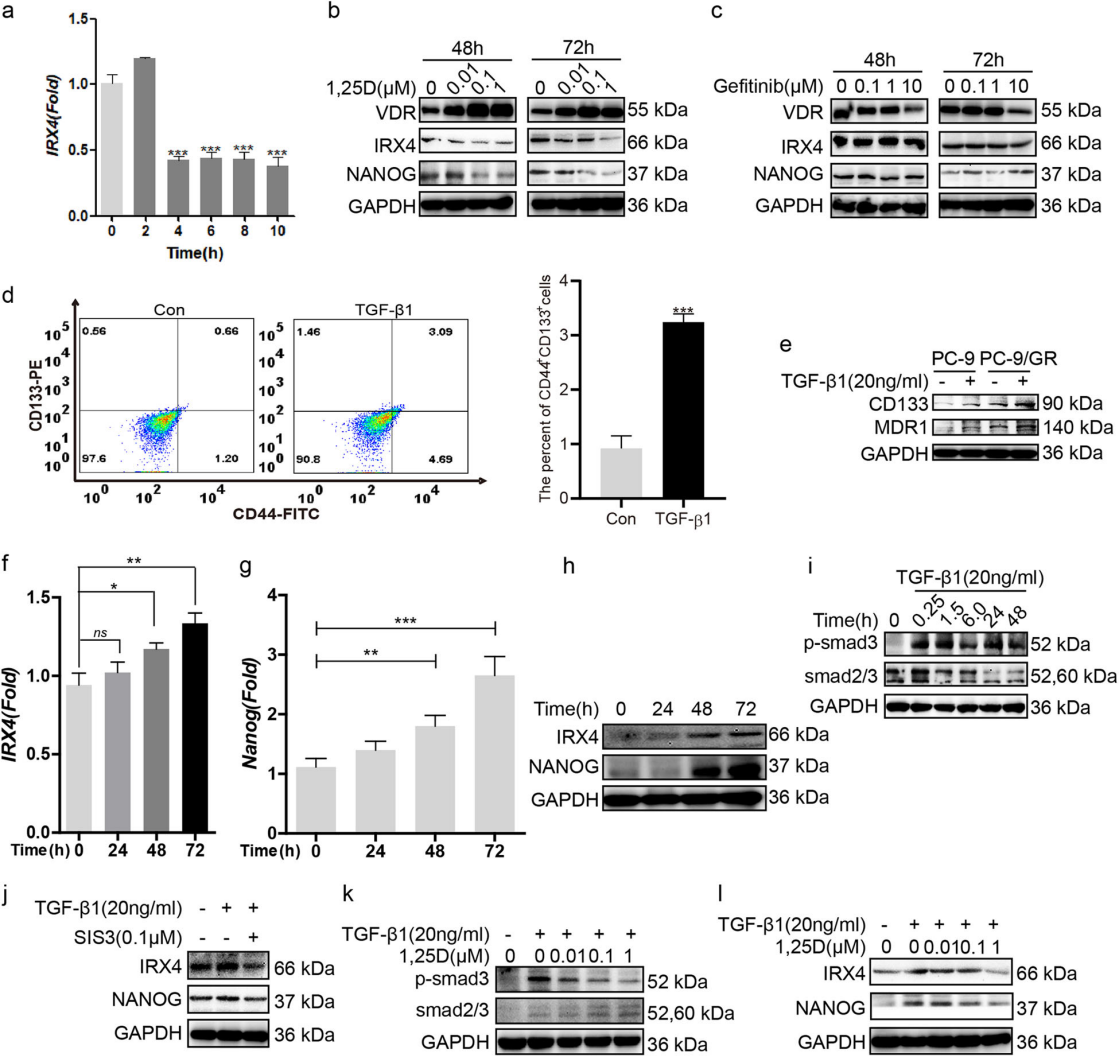
1,25D reduces IRX4 and NANOG expression by inhibiting TGF-β1/Smad3 signaling pathway. **a** The ability of 1,25D reducing *IRX4* transcript at different time point was evaluated by QRT-PCR in PC-9/GR cells (mean ± SD; *n* = 6; ***P < 0.001). **b**, **c** The PC-9/GR cells was treated with 1,25D or gefitinib respectively for 48 h and 72 h. The expression of VDR, IRX4 and NANOG was determined by western blotting. **d** The percentage of CD44^+^CD133^+^ PC-9/GR cells was analyzed by flow cytometry after stimulated with TGF-β1 (20 ng/mL) for 48 h (mean ± SD; *n* = 3; ***P < 0.001). **e** The PC-9 and PC-9/GR cells were respectively stimulated with or without TGF-β1 (20 ng/mL) for 48 h, the expression of CD133 and MDR1 was detected by western blotting. **f**–**h** PC-9/GR cells were stimulated with TGF-β1 (20 ng/mL) at indicated time points, and the expression of IRX4 and NANOG was detected by QRT-PCR or western blotting. **i** PC-9/GR cells were stimulated with TGF-β1 (20 ng/mL) at indicated time points, the expression of p-smad3 and smad2/3 was determined by western blotting. **j** PC-9/GR cells were incubated with SIS3 (0.1 μM) or with their vehicle, 0.1% dimethyl sulfoxideas a control, for 1 h, and were then treated with TGF-β1 (20 ng/mL) for 48 h, the expression of IRX4 and NANOG was evaluated by western blotting. **k**, **l** PC-9/GR cells were pre‐treated with different concentrations of 1,25D for 4 h and then costimulated with 20 ng/mL TGF-β1 for 48 h. Effects of 1,25D on protein levels of p-smad3, smad2/3, IRX4 and NANOG were detected by western blotting.

1,25D downregulated the transcript and protein of IRX4, increased the VDR expression and reduced NANOG expression (Fig. 6a, b). However, gefitinib treatment alone did not significantly change the expression of IRX4 and NANOG, and high dose of gefitinib reduced obviously VDR expression (Fig. 6c). TGF-β1 induced the production of CD44^+^CD133^+^ PC-9/GR cells (Fig. 6d) and increased the expression of CD133 and MDR1 both in PC-9 and PC-9/GR cells (Fig. 6e), moreover, TGF-β1 increased the transcript and protein of IRX4 and NANOG in a time-dependent manner (Fig. 6f–h). TGF-β1 activated Smad3 signaling pathway (Fig. 6i) and the inhibition of TGF-β1/Smad3 signaling with SIS3 reduced obviously the expression of IRX4 and NANOG (Fig. 6j). 1,25D inhibited the activation of Smad3 signaling (Fig. 6k), and the expression of IRX4 and NANOG induced by TGF-β1 in a dose-dependent manner (Fig. 6l).

### 1,25D/gefitinib combination results in increased growth suppression of PC-9/GR cells

**Fig. 7:**
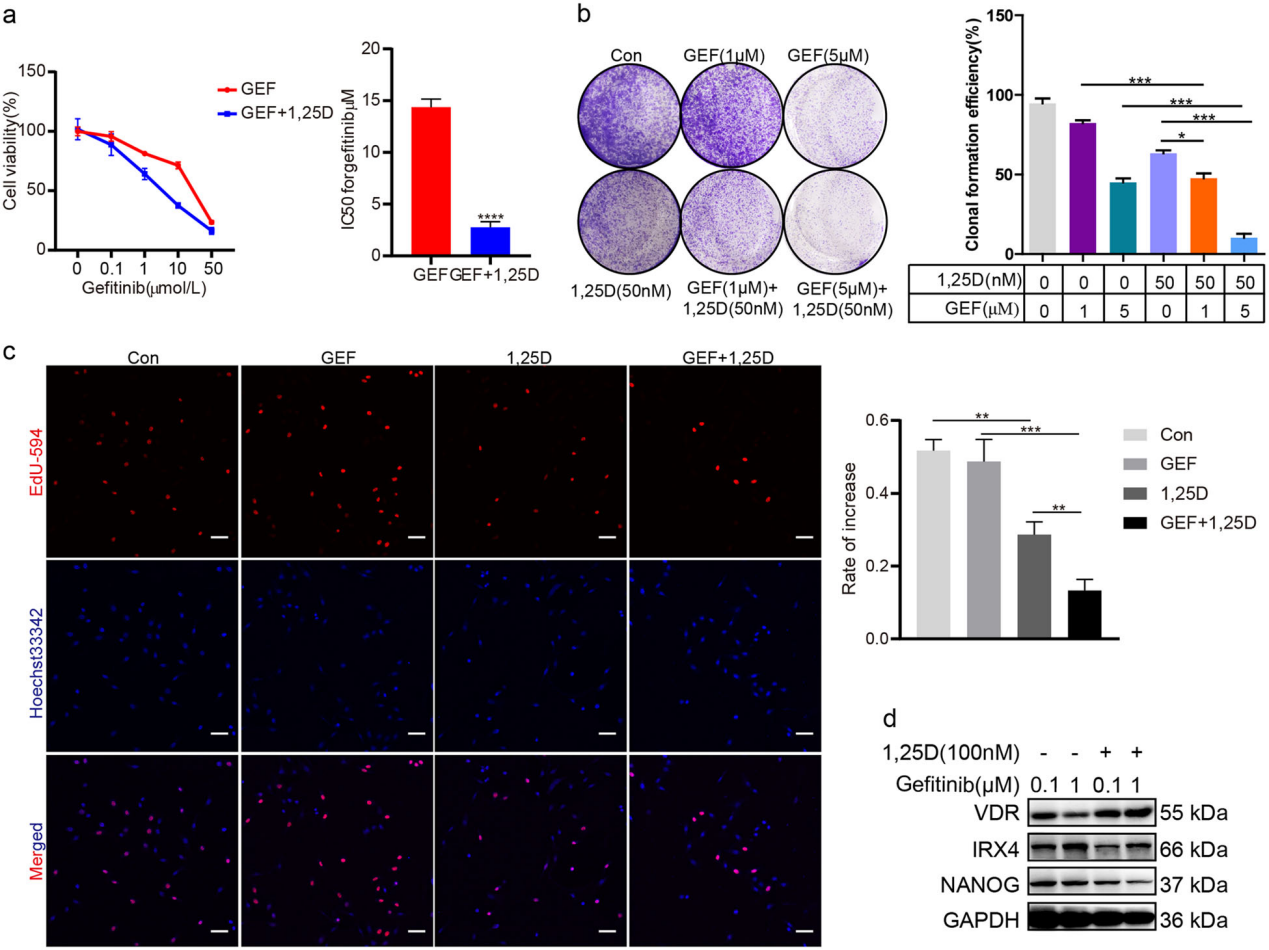
Combined treatment with 1,25D and gefitinib exhibits increased proliferation suppression of PC-9/GR cells. **a** Cells were exposed to various concentrations of gefitinib and gefitinib + 1,25D (100 nM) for 2 days. Cell proliferation was determined by the MTT assay. IC50 value against gefitinib was analyzed. Data represent the mean ± SD of three replicate determinations. ****P < 0.0001. **b** PC-9/GR cells were seeded into 6-well plates (*n* = 3 per group). The next day, the cells were treated with vehicle (control), 1,25D alone (50 nM), gefitinib alone (1 μM and 5 μM), or the combination. Treatments were repeated every 3 days. Colony formation was assessed by crystal violet staining. The colony numbers were assessed with ImageJ software, and the clonal formation efficiency was calculated (mean ± SD; *n* = 3; *P < 0.05, ***P < 0.001). **c** PC-9/GR cells were exposed to vehicle, 1,25D (100 nM), gefitinib (2 μM) or 1,25D + gefitinib for 48 h and stained for Edu (Scale bar: 100 μm; original magnification: ×100; representative images from three experiments). The cell proliferation rate was calculated as a percentage of Edu-positive nuclei to total nuclei (mean ± SD; *n* = 3; **P < 0.01, ***P < 0.001). **d** PC-9/GR cells were exposed to gefitinib (0.1 μM and 1 μM) or 1,25D + gefitinib for 48 h and western blotting was performed to detect the expression of IRX4 and NANOG.

In contrast with gefitinib or 1,25D treatment alone, combined treatment significantly decreased the IC50 value against gefitinib, number of colonies, cells proliferation rate (Fig. 7a–c). Moreover, the expression of VDR was obviously upregulated and the expression of IRX4 and NANOG was significantly downregulated in the combined treatment group than in the gefitinib alone treatment group (Fig. 7d).

### 1,25D/gefitinib combination results in increased growth suppression in vivo

**Fig. 8:**
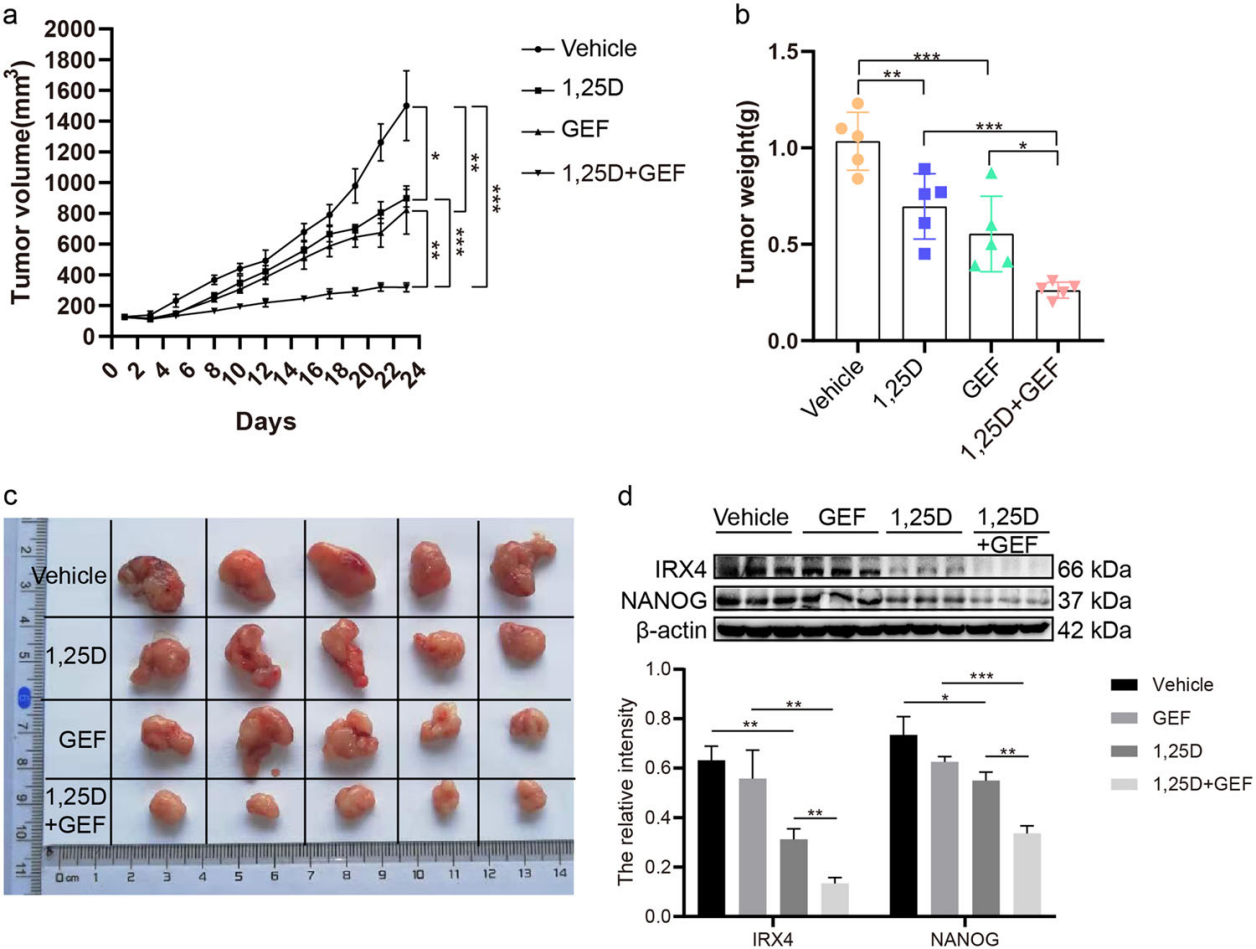
1,25D combined with gefitinib exhibits increased growth suppression in a PC-9/GR tumor xenograft model. **a**–**b** Tumor growth curve and tumor weight for each group were obtained (mean ± SD; *n* = 5; *P < 0.05, **P < 0.01, ***P < 0.001). **c** Macroscopic appearance of PC- 9/GR xenografts of each group. **d** Whole protein cell lysates were prepared randomly from three tumors per group for western blotting to detect the expression of IRX4 and NANOG (mean ± SD; *P < 0.05, **P < 0.01. ***P < 0.001).

Tumors treated with 1,25D and gefitinib alone inhibited tumor progression relative to tumors treated with the vehicle, and treatment with the combination of the two agents significantly suppressed tumor progression compared with one agent alone (Fig. 8a–c). 1,25D treatment obviously decreased the expression of IRX4 and NANOG, and the combined 1,25D and gefitinib treatment clearly reduced IRX4 and NANOG expression compared to gefitinib or 1,25D treatment alone (Fig. 8d). The in vivo results confirm that 1,25D synergizes with gefitinib to inhibit xenograft growth, consistent with the in vitro results.

## Discussion

In this study, we investigated the relationship between IRX4 and gefitinib resistance in NSCLC cell lines, which has not been reported. The results showed that IRX4 expression in PC-9/GR cells with acquired resistance to gefitinib was evidently upregulated compared with that in gefitinib-sensitive PC-9 cells. Moreover, IRX4 expression in H1975 cells with primary resistance to gefitinib was also higher than that in gefitinib-sensitive HCC827 cells. Of note, IRX4 expression has been increasing during the process of PC-9 cells exposing to gefitinib. Next, by silencing IRX4 in PC-9/GR cells, we observed that downregulation of IRX4 inhibited cell viability and the combination of knocking-down IRX4 with gefitinib further decreased cell viability and the level of p-EGFR compared with the single treatment. Our results provide evidence that inhibition of IRX4 expression increases gefitinib cytotoxicity.

How IRX4 controls gefitinib cytotoxicity? Several research groups have observed stem cell-like features, including overexpression of putative stem cell markers ALDH1A1, ABCB1, NANOG, and Sox2 in cells with acquired resistance to gefitinib, afatinib or osimertinib^8,11,21,22^. Furthermore, when the stem cell-like characteristics of gefitinib-resistant cells were lost, gefitinib sensitivity regained^21,23^. These studies have prompted us to investigate whether inhibition of IRX4 expression may increase gefitinib cytotoxicity through regulating stem cell-like properties. We first found that IRX4 knockdown significantly downregulated the pulmosphere forming ability, the percentage of CD133 positive population, and NANOG, Notch1, Sox2, ALDH1A1, and CD133 expression in PC-9/GR cells. Moreover, IRX4 overexpression in PC-9 cells obviously enhanced protein levels of NANOG, CD133 and MDR1. Considering the significant effect of IRX4 regulating NANOG, the Luciferase Reporter Assay was performed, and the results showed that IRX4 could regulate the transcription of NANOG. Further, combined use of gefitinib and IRX4 knockdown significantly downregulated NANOG protein level in vitro, and markedly decreased tumor growth and the expression of NANOG and CD133 in vivo, than treatment with gefitinib alone.

NANOG is a master transcription factor that regulates stem cell pluripotency and cellular reprogramming. RNAi-mediated NANOG knockdown leads to attenuated cancer stem cell (CSC) properties such as sphere formation and clonogenic efficiency in breast and prostate cancer cells^24^. Of note, the protein level of NANOG in lung cancer tissues was upregulated compared to the normal lung tissues and NANOG overexpression predicted a worse prognosis for lung cancer patients^25,26^. Despite the importance of NANOG in lung cancer progression and in regulating CSC properties, the relevance between NANOG with EGFR-TKI resistance is yet unclear. In the current study, the effect of NANOG on regulating gefitinib resistance in PC-9/GR cells was determined by inhibiting NANOG expression via using BBI503 or transfecting siRNA. Results from the experiments showed that decrease of NANOG inhibited CD133 expression and cell proliferation, and enhanced gefitinib cytotoxicity. Our results, together with those previous studies, showed that NANOG was associated with stem-like properties, further indicated that inhibition of NANOG-mediated stem cell-like properties enhanced gefitinib cytotoxicity. Of note, NANOG overexpression-induced cancer stem-like properties and gefitinib resistance could be obviously reversed by knocking-down IRX4. The above results suggest that the regulation of IRX4 on cancer stem-like properties and gefitinib resistance was mediated by NANOG.

How to regulate IRX4-mediated cancer stem-like properties and gefitinib resistance? 1,25(OH)_2_D_3_/VDR signaling pathway restrains cancer stem cell-like properties^19,27^. Based on the observations that inhibition of IRX4 decreased NANOG-mediated cancer cell stemness, we examined the relationship between 1,25(OH)_2_D_3_/VDR and IRX4-induced NANOG expression. We uncovered that 1,25(OH)_2_D_3_ reduced significantly the expression of IRX4 and NANOG. Conversely, gefitinib alone did not exert obvious effect on the IRX4 and NANOG expression. However, when gefitinib was combined with 1,25(OH)_2_D_3_, its ability to decrease IRX4 and NANOG expression was increased. The association between 1,25(OH)_2_D_3_ and TGF-β/Smad signaling pathway^28,29^ led us to consider whether TGF-β/Smad signaling pathway induces the IRX4-induced cancer stem cell-like properties. Studies have shown that TGF-β1 signaling promotes cancer cell stemness, leading to tumor metastasis^30,31^. Whether TGF-β1 increased cancer cell stemness by regulating IRX4 and how to regulate TGF-β1 signaling mediated cancer stem-like properties is unclear. In this study, we discovered that TGF-β1/Smad3 signaling increased IRX4 expression and NANOG-mediated cancer cell stemness. Notably, high TGF-β1 levels were correlated with poor EGFR-TKI sensitivity and overall survival in NSCLC samples. Moreover, TGF-β1 could increase EGFR-TKI resistance in PC-9 cell lines^32^ and in A549 cells^33^. Therefore, we speculate that TGF-β1 may increase EGFR-TKI resistance by regulating IRX4-induced cancer stem-like properties. Next, whether 1,25(OH)_2_D_3_ regulates the TGF-β/Smad signaling-induced IRX4 and NANOG expression or is simply correlated with it, was determined. The results demonstrated that 1,25(OH)_2_D_3_ inhibited the activation of Smad3, and the ability of TGF-β1 to induce IRX4 and NANOG expression was attenuated by 1,25(OH)2D3. Furthermore, we discovered that, both in vitro and in vivo, combination of 1,25(OH)_2_D_3_ with gefitinib resulted in significant growth inhibition of PC-9/GR cells, and combination treatment further decreased the expression of IRX4 and NANOG compared with single treatment. In light of our new data, we conclude that 1,25(OH)_2_D_3_ signaling-induced decreases in IRX4 inhibits NANOG-mediated cancer stem-like properties and gefitinib resistance in PC-9/GR cells.

In summary, the roles of IRX4 downregulation increasing gefitinib cytotoxicity and inhibiting NANOG-mediated cancer stem-like properties were elucidated for the first time, to the best of our knowledge. Furthermore, collective efforts have been done to explore how to regulate IRX4 and NANOG expression and to find new strategies overcoming gefitinib resistance, such as, combination of gefitinib with 1,25(OH)_2_D_3_. Our study provides a new strategy through targeting IRX4 to enhance gefitinib cytotoxicity.

## Materials and methods

### Cell lines

NSCLC lines PC-9 and PC-9/GR (The gifts from Nanjing Medical University), A549 (ATCC^®^ CCL-185™, ATCC, Manassas, VA, USA), HCC827 (ATCC^®^ CRL-2868™), H1975 (ATCC^®^ CRL-5908™), H1650 (ATCC^®^ CRL-5883™) and H23 (ATCC^®^ CRL-5800™) were cultured in Dulbecco modified Eagle medium (DMEM) (Biological Industries, Kibbutz Beit-Haemek, Israel) or RPMI 1640 (Biological Industries) supplemented with 10% fetal bovine serum (FBS) (Biological Industries), 100 μg/mL streptomycin and 100 U/mL penicillin (KeyGEN BioTECH, Nanjing, China) in a humidified cell incubator at 37 °C with an atmosphere of 5% CO_2_.

### Reagents and chemicals

The reagents used in this study were purchased as follows. Primers were ordered from Genscript (Nanjing, China). Gefitinib, BBI503, SIS3, and 1,25(OH)_2_D_3_ (calcitriol) were purchased from selleck (Shanghai, China). TGF-β1, EGF and FGF-basic were obtained from PeproTech (Rocky Hill, NJ, USA). B27 was obtained from Gibco (Gaithersburg, MD, USA).

### Western blot analysis

Proteins were extracted from the NSCLC cells (*n* = 3, biological replicates) or tumor tissues in the lysis buffer (KeyGEN BioTECH, Nanjing, China) and protease inhibitor cocktail (KeyGEN BioTECH, Nanjing, China) for western blotting^34^. Antibodies used for western blotting were: anti-IRX4 (ab123542, Abcam, Cambridge, UK), anti-pEGFR, EGFR, p-smad3, smad2/3 (#3777, #4267, #9520, #8685, Cell Signaling Technology, Boston, MA, USA), anti-CD133, NANOG, Notch1, β-catenin, MDR1, VDR, ABCG2 and anti-Histone-H3 (18470-1-AP, 14295-1-AP, 20687-1-AP, 51067-2-AP, 22336-1-AP, 14526-1-AP, 27286-1-AP, 17168-1-AP, Proteintech group, WUHAN SANYING, WuHan, China), anti-Sox2, Oct4, ALDH1A1 (D164316, D121072, D220058, Sangon Biotech, Shanghai, China), and anti-β-actin, anti-GAPDH (bs-0061R, bs-0755R, Bioss, Beijing, China) antibodies, HRP-conjugated goat anti-rabbit IgG secondary antibody (ABL3012-2, AbSci, Vancouver, WA, USA). The electrochemical luminescent substrates (Tanon, Shanghai, China) were used according to the manufacturer’s protocol in order to visualize the proteins of interesting in the Tanon imaging system. The relative expressions were quantified densitometrically using the ChemiScope analysis software and calculated according to the reference bands of GAPDH or β-actin.

### Quantitative real-time PCR

Total RNA was extracted from NSCLC cells using Trizol reagent (Vazyme, Nanjing, China) as described by the manufacturer before cDNA synthesis (Vazyme, Nanjing, China) (*n* = 3, biological replicates). The level of GAPDH RNA expression was used to normalize the data. The mRNA primer sequences (GenScript, Nanjing, China) used for QRT-PCR were human IRX4: 5′-CCTTCTACTCGCTGAACA-3′ (forward, F), 5′-TGGCTCGTAAGGGTAGTA-3′(reverse, R); human NANOG: 5′-CTCCAACAT CCTGAACCT-3′ (F), 5′-GTCACAC CATTGCTATTCTT-3′ (R); human CTNNB1: 5′-CAAGCCACAAGATTACAAGA-3′(F), 5′-CACCAATATCAAGTCCAAGAT-3′ (R); human Notch1: 5′-GGACCTCATCAACTCACA-3′(F), 5′-TTCTTCAGGAGC ACAACT-3′ (R); human IRX3: 5′-CTGTAGTGCCTTGGAAGT-3′(F), 5′-GGAGAG AGCCGATAAGAC-3′ (R); human IRX5: 5′-CCTATCCGCAGGGCTACTTG-3′(F), 5′-AATGACGCTGGTGCTGTACG -3′ (R); human GAPDH: 5′-CTTCTTTTGCGTC GCCAGCCGA-3′(F), 5′-ACCAGGCGCCCAATACGACCAA-3′ (R); Primers were used to quantify expression with RT-PCR (Applied Biosystems, Foster City, CA, USA) and results were analyzed with the ΔΔCt method.

### Cell viability assays and Edu staining

The cell viability assay was performed as previously described^35^. Briefly, 2 × 10^3^ cells/well was seeded in 96-well plates (*n* = 6 per group, *n* = 3, biological replicates). Twenty-four hours after seeding, the cells were transfected with siRNA, or treated with various concentrations of gefitinib or gefitinib + 1,25(OH)_2_D_3_ in medium containing 10% FBS for 48 h. Absorbance was measured at indicated time points. Cell proliferation was quantified based on the incorporation of 5-ethynyl-2’-deoxyuridine (Edu) into DNA using a BeyoClick™ EdU-594 In Vitro Imaging Kit (Beyotime, Nantong, China) as previously described^36^. A laser scanning confocal microscope (Carl Zeiss, Jena, Germany) was used to determine the proportion of nucleated cells that had incorporated Edu. The assay was performed in triplicate and repeated three times in independent experiments.

### Cell HE staining

The cells were seeded in 6-well plates. Twenty-four hours after seeding, the cells were washed by PBS for three times and fixed with methanol for 5 min, and then stained with hematoxylin and eosin (*n* = 2, biological replicates). The cell morphology was recorded by microscopy (Olympus, Tokyo, Japan).

### siRNA and plasmid transfection

In all experiments, 150 pmol siRNA (the target sequence of IRX4‐specific siRNA: 5′‐GGAAAUGUCUCCAUUAGUUTT‐3′ or NANOG-specific siRNA: 5′-GGA GGUCCUAUUUCUCUAATT-3′) (TranSheep Bio, Shanghai, China) or 5 μg of pcDNA3.1‐IRX4 or pcDNA3.1-NANOG or empty vector (HanBio, Shanghai, China) were used to transfect 70–80% confluent cells according to the manufacturer’s instructions (*n* = 3, biological replicates). The Lipofectamine 2000 reagent (Life Technologies, Carlsbad, CA, USA)was used to deliver siRNA or plasmid into PC-9/GR or PC-9 cells growing in serum‐free opti‐MEM media (Gibco, Gaithersburg, MD, USA). After 6 h, the medium containing the siRNA/plasmid‐lipid complexes was replaced with DMEM containing 10% FBS. And subsequent experiments were completed at indicated time after transfection.

### Luciferase reporter assay

pGL3-NANOG-luc (pNANOG-luc) was obtained from HanBio (Shanghai, China). The reporter constructs containing 2331 bp (-2309 to 22) of NANOG promoter. PC-9/GR cells were transfected with the siRNA targeting IRX4 or negative control and the pNANOG-luc plasmid, and the pRL-TK was co-transfected in each experiment as an internal control for transfection efficiency. At 48 h post-transfection, cells were lysed and the luciferase activities were measured using the Dual-Luciferase Reporter Assay System (Vazyme, Nanjing, China) and a Multifunctional microplate reader (Molecular Devices ID5, Sunnyvale, CA, USA). The firefly luciferase activity was corrected by the corresponding Renilla luciferase activity and the experiments were carried out in triplicate.

### Colony-formation assay

The cells were stimulated with various concentration of gefitinib or transfected with siCtrl or siIRX4 or siNANOG. After approximately 14 days of culturing, the colonies were fixed in methanol and stained with 0.1% crystal violet (KeyGEN BioTECH, Nanjing, China). Colonies with a diameter greater than 1 mm were counted. Samples were assayed in triplicate.

### Lentivirus infection

1.5 × 10^5^ PC-9/GR cells were seeded in 6-well plates. Twenty-four hours after seeding, the cells were treated with polybreen (5 μg/mL) and respectively infected with various volume of lentivirus (4 × 10^8^TU/mL) carrying shNC or shIRX4 (HanBio, Shanghai, China). After 72 h, the cells were collected, and ZsGreen positive cells were analyzed by flow cytometry (Miltenyi Biotec, Cologne, Germany) (*n* = 2, biological replicates) and FlowJo VX software, and the expression of IRX4, NANOG, Sox2, Oct4, ABCG2, and ALDH1A1 was evaluated by western blotting (*n* = 3, biological replicates).

### Flow cytometry

The experiment was performed as methods described previously^37^. Briefly, 1 × 10^6^ cells was washed twice with PBS supplemented with 0.5% BSA and 2 mM EDTA and incubated with 5 μL each of anti-CD133-PE (TMP4 clone; eBioscience, Waltham, MA, USA) or anti-CD44-FITC (BJ18 clone; BioLegend, San Diego, CA, USA) antibodies diluted in 100 μL of staining solution for 15 min at room temperature. Then, after washing the cells twice, 400 μL of buffer was added, and samples were analyzed by flow cytometry (Miltenyi Biotec) and FlowJo VX software (*n* = 3, biological replicates).

### Xenografts assay

Four-week-old male BALB/c nude mice were obtained from Cavens Experimental Animal Co., Ltd (Changzhou, China). All experiments involving animals were approved by the Ethics Committee of China Pharmaceutical University. The animals were maintained in individual ventilated cages in compliance with institutional guidelines. The xenograft mouse model was established as methods described previously^38^. Briefly, the PC-9/GR cells were respectively infected with a lentivirus carrying control shRNA (shNC) or with shIRX4 (Contract number HH20190622LHY-LV03, HanBio, Shanghai, China). After 3 days, the cells were harvested and resuspended respectively, then approximately 1.5 × 10^7^ cells were subcutaneously injected into the right forelimb of mice. The mice of shNC and shIRX4 group were randomized into four groups (*n* = 8 per group), and when tumor volume reaches an average of 80mm^3^, the mice were treated with gefitinib (50 mg/kg) or drinking water (vehicle) alone. In addition, another xenograft mouse model was established, and the effect of 1,25(OH)_2_D_3_ combined with gefitinib was evaluated. Briefly, approximately 1 × 10^7^ PC-9/GR cells were subcutaneously injected into the right forelimb of mice. Treatment began 1 week following injection. The mice were randomized into four groups (*n* = 8 per group) and intragastric administrated with vehicle (PBS), 1,25(OH)_2_D_3_ (1 μg/kg/2day), gefitinib (50 mg/kg/day) or 1,25(OH)_2_D_3_ (1 μg/kg/2day) + gefitinib (50 mg/kg/day). The tumor volume was measured once every two days and calculated as (length×width^2^)/2. The animals were monitored for approximately 3 weeks until euthanasia. Mice died before being sacrificed were excluded, and the xenografts from each group were collected for further analyses. No blinding was carried out for animal experiments.

### Statistical analysis

Data were expressed as mean values ± Standard Deviation. Power analysis was performed to choose the sample size. Two-sided statistical tests were performed. Unpaired Student’s *t* test was used when comparing two groups. One-way ANOVA with Dunnett’s test or two-way ANOVA with Bonferroni’s multiple comparison was used to compare multiple groups. Statistical analysis was performed using Prism 8.00 software (GraphPad, San Diego, CA, USA). The differences were considered significant for *P* < 0.05.
